# Multi-antigen Vaccination With Simultaneous Engagement of the OX40 Receptor Delays Malignant Mesothelioma Growth and Increases Survival in Animal Models

**DOI:** 10.3389/fonc.2019.00720

**Published:** 2019-08-02

**Authors:** Peter R. Hoffmann, Fukun W. Hoffmann, Thomas A. Premeaux, Tsuyoshi Fujita, Elisa Soprana, Maddalena Panigada, Glen M. Chew, Guilhem Richard, Pooja Hindocha, Mark Menor, Vedbar S. Khadka, Youping Deng, Lenny Moise, Lishomwa C. Ndhlovu, Antonio Siccardi, Andrew D. Weinberg, Anne S. De Groot, Pietro Bertino

**Affiliations:** ^1^Department of Cell and Molecular Biology, John A. Burns School of Medicine, University of Hawai'i, Honolulu, HI, United States; ^2^Department of Tropical Medicine, John A. Burns School of Medicine, University of Hawai'i, Honolulu, HI, United States; ^3^Department of Molecular Immunology, San Raffaele University and Research Institute, Milan, Italy; ^4^EpiVax, Inc., Providence, RI, United States; ^5^Bioinformatics Core, Department of Complementary and Integrative Medicine, John A. Burns School of Medicine, University of Hawai'i, Honolulu, HI, United States; ^6^Department of Cell and Molecular Biology, Institute for Immunology and Informatics, University of Rhode Island, Providence, RI, United States; ^7^Robert W. Franz Cancer Research Center, Earle A. Chiles Research Institute, Providence Portland Medical Center, Portland, OR, United States

**Keywords:** cancer vaccines, OX40, mesothelioma, epimatrix, immunotherapy

## Abstract

Malignant Mesothelioma (MM) is a rare and highly aggressive cancer that develops from mesothelial cells lining the pleura and other internal cavities, and is often associated with asbestos exposure. To date, no effective treatments have been made available for this pathology. Herein, we propose a novel immunotherapeutic approach based on a unique vaccine targeting a series of antigens that we found expressed in different MM tumors, but largely undetectable in normal tissues. This vaccine, that we term p-Tvax, is comprised of a series of immunogenic peptides presented by both MHC-I and -II to generate robust immune responses. The peptides were designed using *in silico* algorithms that discriminate between highly immunogenic T cell epitopes and other harmful epitopes, such as suppressive regulatory T cell epitopes and autoimmune epitopes. Vaccination of mice with p-Tvax led to antigen-specific immune responses that involved both CD8^+^ and CD4^+^ T cells, which exhibited cytolytic activity against MM cells *in vitro*. In mice carrying MM tumors, p-Tvax increased tumor infiltration of CD4^+^ T cells. Moreover, combining p-Tvax with an OX40 agonist led to decreased tumor growth and increased survival. Mice treated with this combination immunotherapy displayed higher numbers of tumor-infiltrating CD8^+^ and CD4^+^ T cells and reduced T regulatory cells in tumors. Collectively, these data suggest that the combination of p-Tvax with an OX40 agonist could be an effective strategy for MM treatment.

## Introduction

Malignant Mesothelioma (MM) is a rare but highly aggressive cancer that develops from mesothelial cells lining the pleura and other internal cavities and is often associated with asbestos exposure. To date, no effective treatments are available for MM (1). Over the past decade, immunotherapeutic approaches have created new opportunities to efficiently combat cancer progression. The introduction of CAR T-cell therapies as well as the successful use of antibody-based inhibitors of immune checkpoints (e.g., Cytotoxic T Lymphocyte Associated protein 4, CTLA-4; Programmed cell Dead protein 1, PD-1; and its ligand PD-L1) have invigorated the field of immunotherapy and benefited an increasing number of cancer patients (2). Anti-tumor therapy using personalized vaccines individually tailored for immune responses against the patient's mutanome have been demonstrated to improve the therapeutic outcome of biologics (3, 4). This approach, however, includes limitations such as potential delays between biopsy, design and production of the vaccine, in addition to high costs for vaccine production. Moreover, MM vaccines containing epitopes that are common to MM may be used as a means to prevent tumor development, for individuals exposed to asbestos and in regions of the world where MM risk is high. MM requires up to 40 years from the time of asbestos exposure to the development of disease, therefore, it is conceivable that preventive vaccination approaches such as the one currently used for HPV-associated cervical cancer might similarly reduce MM incidence.

Herein, we describe the development of a novel immunotherapeutic approach based on a unique vaccine that targets a series of self-antigens that we found to be commonly over-expressed in different MM mouse tumors compared to normal tissues. Efficacious treatment using this vaccine is demonstrated in three different MM animal models, suggesting this approach may be used as a model to develop an off-the-shelf immunotherapy for human patients that overcomes some of the limitations of personalized anti-cancer vaccines described above. Our vaccine, named p-Tvax, is comprised of a series of immunogenic peptides containing both MHC-I and -II restricted T cell epitopes and derived from multiple MM antigens. The antigens included in the vaccine were chosen based on their high expression in MM tumor tissues and low levels in normal tissues in an attempt to minimize the risk of triggering immune responses against healthy tissues. Each peptide was designed using the iVAX platform comprising of state-of-the-art *in silico* algorithms including EpiMatrix and JanusMatrix, which have been previously demonstrated as effective tools to discriminate between highly immunogenic T cell epitopes and other undesirable epitopes such as suppressive regulatory T cell epitopes (Tregitopes) or autoimmune epitopes (autoepitopes) (5–7). JanusMatrix's ability to identify tumor epitopes cross-conserved with autoepitopes is particularly relevant for designing new cancer immunotherapies, as exemplified by previous therapies failing due to off-target cardiac or neurologic toxicities (8, 9).

Even if peptide-based vaccines are found to activate anti-cancer T cells, the efficacy of these immune cells is mitigated within the tumor microenvironment by several mechanisms. Many of these suppressive mechanisms are driven by immunological checkpoint molecules such as PD-1 and CTLA-4, or by immune co-stimulatory proteins such as OX40. To overcome these suppressive mechanisms and generate effective anti-tumor immune responses, we have combined p-Tvax with an OX40 agonist. OX40 is a Tumor Necrosis Factor receptor family member that is expressed by both activated T effector cells and Foxp3^+^ T regulatory cells (Tregs). Ligands that promote OX40 signaling, as well as agonistic monoclonal antibodies (mAbs) that target this molecule, induce the activation and proliferation of effector T cells, while reducing Treg activity through the inhibition of Foxp3 gene expression (10–12). Humanized versions of OX40 agonists have been positively evaluated in a phase I clinical trial, and are now under investigation in phase II (13). In this study, we present results of this dual therapeutic approach that support the concept that a universal cancer vaccine for MM may offer a safe and potentially curative therapy for this deadly cancer when combined with mAbs that target T cell co-stimulation.

## Materials and Methods

### Mice and Cells

Female 6–8 week-old BALB/c mice were obtained from the Jackson Laboratory. Animal experiments were performed in accordance with institutional guidelines and approved by the University of Hawaii IACUC (#16-2355). Murine AB12 MM cells derived from asbestos-induced tumors in a BALB/c mouse were provided by Dr. B. Robinson (University of Western Australia, Nedlands, Australia) (14). Murine CRH5 and EOH6 MM cells were isolated from peritoneal ascites developed in asbestos- or erionite-injected mice in carcinogenesis experiments as previously described (15). Mesothelial cells were isolated as previously described from naive BALB/c mice (16). All cells were cultured in Ham's F12 medium (Corning) containing 10% fetal bovine serum (FBS) and antibiotics. All MM cells used in this study were provided to our laboratories or purchased between 2004 and 2007.

### Transcriptome Microarray Analysis

BALB/c mice were injected subcutaneously (s.c.) with 10^5^ of either CRH5 or EOH6 MM cells. When tumors reached 100 mm^3^ they were excised and total RNA was extracted. At the same time, RNA was isolated from lungs and kidneys excised from naïve BALB/c mice. RNA expression in the different tissues was evaluated using the Clariom S Mouse Array (Affymetrix). Expression values were normalized and summarized into transcript clusters for analysis using Robust Multi-array Average approach in Array Studio (OmicSoft, Cary, NC). One-way ANOVA was used to look for differential expression between normal and tumor samples, and *p*-values were adjusted for multiple comparisons using the Benjamini-Hochberg False Discovery Rate (FDR) method. Only candidates with FDR-adjusted *p* < 0.001 were considered. The data gathered from this analysis were deposited in the Gene Expression Omnibus (GEO) database (Accession Number: GSE122004).

### Western Blot Analysis

Frozen tumors and normal tissues were lysed in ice-cold buffer containing 150 mM NaCl, 50 mM Tris, 1% Triton X-100, 1% sodium deoxycholate, and protease inhibitor cocktail (Roche Applied) at 4°C for 1 h. Insoluble material was removed by centrifugation at maximum speed for 5 min, and total protein in the supernatant was determined using a Bradford assay reagent (Bio-Rad). After adjusting to equal protein concentration, lysates were boiled in SDS sample buffer and then separated by SDS-PAGE, followed by transfer of the proteins onto nitrocellulose membranes. Blots were incubated with primary anti-TROAP (Clone 3-11, Novus Biological), anti-OLFML2B (Mybiosurce), anti-KIF20A (Clone D-3 Santa Cruz), or anti-β-actin (Sigma) for 1.5 h, washed, incubated with appropriate HRP-conjugated secondary antibody (1:20,000; Li-Cor), and visualized using the Odyssey Scanner (Li-Cor).

### Epitope Selection and Peptide Synthesis

Antigens selected after the transcriptomic analysis were screened for BALB/c MHC-I (H2-Dd, H2-Kd) and MHC-II (H2-IAd, H2-IEd) restricted T cell epitopes using the EpiMatrix algorithm. In addition, putative Tregitopes and autoepitopes were identified using the JanusMatrix algorithm (6). Briefly, each protein sequence was first parsed into overlapping 9-mer frames. Each frame was then evaluated with EpiMatrix and JanusMatrix to determine its likelihood of binding to MHC-I (H2-Dd, H2-Kd) and MHC-II (H2-IAd, H2-IEd) alleles and its potential to induce Tregs, respectively. One peptide sequence enriched in MHC-I and -II epitopes and devoid of putative Tregitopes was derived from each selected antigen. Synthetic peptides were manufactured by twenty-first century Biochemicals (Marlboro, MA) using fluorenylmethoxycarbonyl chemistry and solid-phase synthesis and purified by high-pressure liquid chromatography. The quality of the peptides was assessed by high-performance liquid chromatography analysis. Peptide purity was >90% as ascertained by analytical reversed phase HPLC. Individual peptides were dissolved with 10 μl DMSO, diluted to 1 mg/ml with PBS and used at a final concentration of 10 μg/ml in *ex vivo* assays (DMSO 0.001%). A mixture of all peptides (10 μg/peptide), which we termed p-Tvax, was used to vaccinate mice in 100 μl PBS.

### Immunotherapies Schedule and Flow Cytometric Analysis for Intracellular IFN-γ

BALB/c mice were vaccinated with two s.c. injections of p-Tvax peptides (10 μg of each of the seven peptides, diluted in PBS), 1 week apart (day 2 and day 9). Two days before each vaccination, 50 μg CpG ODN 1585 adjuvant (Invitrogen) was injected at day 0 and day 7, while 200 μg of OX40 agonists (clone OX86, kindly provided by Dr. A. Weinberg) were injected at day 4 and day 9. Five days after the last vaccination, spleens were excised from controls, and from mice treated with the different immunotherapies. One million cells were stimulated for 24 h with 10 μg/ml of each p-Tvax peptides. For detection of IFN-γ in the cytoplasm, Brefeldin A was added 6 h before staining. Fixation/permeabilization kits were used in combination with the following fluorochrome conjugated monoclonal antibodies: Anti-CD3-PerCP/Cy5.5 (clone OKT3), anti-CD4-AlexaFluor700 (clone RM4-5), anti-CD8-APC/Cy7 (clone 53-6.7), and IFN-γ-FITC (clone XMG1.2) antibodies. Live cells were distinguished from debris using Aqua LIVE/DEAD^®^ cell viability dye (all from Biolegend). Cells were evaluated using LSRFortessa Flow Cytometer (BD Biosciences) and the data were analyzed with FlowJo software.

### Granzyme B ELISpot Assay

To measure T cell cytotoxicity, granzyme B secretion was analyzed using the Mouse Granzyme B ELISpot Kit (R&D system). Splenocytes were cultured with each single p-Tvax peptide for 6 d, with peptides replenished every 2 d. At day 3, cultures were supplemented with 5 IU/ml IL-2. At day 6, splenocytes stimulated with the different peptides were pooled together and dead cells removed with Lympholyte M (Cedarlane). Splenocytes not stimulated with the peptides were processed similarly. Stimulated and control splenocytes (10^4^ effector cells) were then co-cultured with different MM cells (5 × 10^3^ target cells) or with mesothelial cells in 200 μL media. Negative controls consisted of effector cells in the absence of target cells, target cells in the absence of effector cells and media only. After 4 h of incubation, detection of granzyme B spots was performed following the manufacturer's directions. Spots were then analyzed and counted with an ImmunoSpot analyzer (CTL), with the instrument sensitivity kept low to reduce background.

### Murine Therapeutic Experiments

To evaluate the efficacy of the different immunotherapies on tumor dimensions and mouse survival, s.c., and intraperitoneal (i.p.) mouse models of MM were employed. In the s.c. model, 5 × 10^4^ CRH5 cells were injected in the hind flank in cohorts of five BALB/c mice. When tumors became palpable on day 7 (3–4 mm in maximal diameter), mice were vaccinated as described above. Tumor size was measured weekly using digital calipers until the first death was recorded. Survival was then followed until tumors reached volumes >300 mm^3^. For the i.p. model of MM, 2 × 10^5^ EOH6 cells or 5 × 10^4^ AB12 cells were injected i.p. in cohorts of 5 BALB/c mice. Both MM cells were previously transduced with the lentiviral vector hPGK.lu2.WPRE.mhCMV.dNGFR.SV40PA, which encoded the bioluminescent genetic marker luciferase (provided by Dr. Naldini, San Raffaele University and Research Institute, Milano, Italy). Immunotherapies were performed as scheduled for the s.c. model. To assess tumor dimension and localization of luminescent cells, mice were injected i.p. with 15 mg/ml d-luciferin, bioluminescence signals of MM inoculated mice were monitored using the IVIS system (PerkinElmer). Regions of interest were identified around the tumor sites and were quantified as total photon counts using Living Image software (PerkinElmer). For survival, animals were monitored weekly and euthanized when they appeared moribund according to IACUC guidelines.

### Isolation and Analysis of Tumor-Infiltrating Immune Cells

CRH5 or EOH6 MM cells were injected s.c. in cohorts of five BALB/c mice. When tumors reached 50 mm in maximal diameter, mice received the same immunotherapies regimens indicated above. Five days after the last vaccination, tumors were excised, washed with PBS, minced and incubated for 1 h at 37°C in digestion buffer consisting of 1 mg/ml collagenase IV, 100 μl/ml hyalurodinase and 15 mg/ml DNAse I (all from Roche Applied Sciences) in PBS. After digestion, tumors were forced through a 40 μm cell strainer. A total of 10^6^ cells were stained for flow cytometer analysis to characterize tumor-infiltrating T lymphocytes. The same fluorochrome conjugated monoclonal antibodies used for the analysis of T cell responses were utilized plus anti-CD25-PE/Cy7 clone PC61 and anti-FoxP3-FITC clone MF-14 for Tregs analysis, or plus anti-PD-1-PE/Cy7 clone RMP1-30 and anti-OX40-PE clone OX86 for T cell marker analysis (all from Biolegend). Cells were analyzed using LSRFortessa Flow Cytometer (BD Biosciences) and data organized with FlowJo software.

### Statistical Methods

All statistical tests were performed using GraphPad Prism 7.0. Means were compared using two-way ANOVA followed by the Bonferroni multiple comparison test. For survival, differences were evaluated using Kaplan-Meier curves with log-rank test. Data are represented as mean ± S.E. with statistical significance values indicated in the figure legends together with the n values used to calculate the statistics. All *in vitro* experiments with MM cells have been repeated at least three times using samples from the same source as technical replicates. *In vivo* studies as well as experiments with primary cells were also repeated at least three times using different sources as biological replicates.

## Results

### Transcriptome Analysis and Antigen Selection

**Figure 1:**
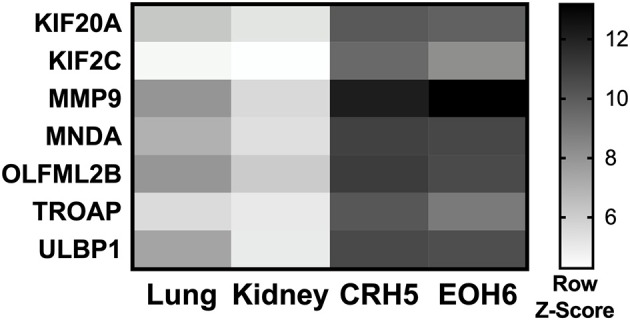
p-Tvax antigens are overexpressed in MM tumors. Transcriptome analysis using mRNA-microarrays was performed on two MM mouse tumors (grown from injected CRH5 or EOH6 cells) and two normal tissues (kidney and lung). mRNA levels are indicated by Row Z-Score colors in a heat map. Antigens with the higher mRNA level for both tumors, compared with both normal tissues, were selected for inclusion in the p-Tvax vaccine. Differences in mRNA levels between each MM tumor and each normal tissue were statistically significant for all antigens (*p* < 0.001).

With the goal of designing a universal vaccine that can be used to target MM as tested in mice, we analyzed expression of all the mRNA produced by different MM tumors and normal tissues. Our hypothesis was that antigens highly expressed in MM tumors can be targeted for vaccination, as long the expression of those antigens is extremely low in all normal mouse tissues. mRNA was analyzed in two tumors originating from two different MM cell lines injected in BALB/c mice, CRH5, and EOH6. These cells were previously generated by injecting asbestos or erionite in the same mouse strain (15). For the normal tissues, we analyzed antigen expression in lungs and kidneys isolated from naïve BALB/c mice. Selected antigens had the highest expression in both MM tumors and the lowest in both normal tissues. Among these, only antigens that were commonly overexpressed by both MM tumors were included in the multi-antigen universal vaccine. Since we analyzed only two normal mouse tissues, we also considered transcriptome studies performed in other studies to evaluate the expression of the selected antigens in normal tissues (17, 18). At the end of this process, we selected the following seven antigens: KIF20A, KIF2C, MMP9, MNDA, OLFML2B, TROAP, and ULBP1. The mRNA expression levels for these antigens in tumor and normal tissues are shown in Figure 1. We also tried to evaluate protein expression of these antigens in CRH5 and EOH6 MM tumors with western blot assays. In these experiments, among the numerous antibodies tested, only those for OLFML2B, TOAP, and KIF20A specifically detected the proteins targeted by the universal vaccine (Supplementary Figure 1).

### *In silico* Epitope Mapping and Peptide Selection

Each of the antigens selected for vaccination was screened for MHC-I and MHC-II T cell epitopes using the EpiMatrix algorithm. Using the JanusMatrix algorithm, we also identified putative regulatory T cell epitopes (Tregitopes), which can promote suppression of vaccine-induced T cell responses, as well as autoimmune epitopes (autoepitopes). For each of the seven antigens, we selected a sequence that contained multiple predicted MHC-I and MHC-II restricted T cell epitopes, and no Tregitopes nor autoepitopes (Table 1). A mixture of all these peptides was used as multi-antigen vaccine named p-Tvax.

**Table 1:** p-Tvax epitopes.

**N**.	**Antigen**	**Start position**	**Peptide**	**Length**
1	KIF20A	860	Ac-SSTDSSPYARILRSRHSPLLK-amide	21
2	KIF2C	356	GDLSGKSQNASKGIYAMASRDVFLLKN-amide	27
3	MMP9	594	RVFFFSGRQMWVYTGKTVLGPRSLDKLGL-amide	29
4	MNDA	298	Ac-NETSSVLEAAPKQMIEVPNCITRN -amide	24
5	OLFML2B	50	DNQENVLSQLLGDYDKVKAVSEGSD-amide	25
6	TROAP	156	Ac-KGGTTQRGQSARSSAYLAPRIPTH-amide	24
7	ULBP1	56	Ac-LNRQPLFVYKDKKCHAIGAHRNSMNATKI-amide	29

*Amino acid sequences of each antigen were screened with EpiMatrix to identify epitopes with potential immune stimulatory properties in BALB/c mice. Putative regulatory T cells epitopes (Tregitopes), which promote suppression of vaccine-induced T cell responses, and autoimmune epitopes (autoepitopes) that have potential of inducing autoimmune reactions were selected against using JanusMatrix algorithm. For each antigen, a peptide that contained both MHC-I and -II epitopes was synthetized as listed and used for vaccination, or for detection of antigen-specific immune responses*.

### p-Tvax Vaccination Induces Antigen-Specific T Cell Responses

**Figure 2:**
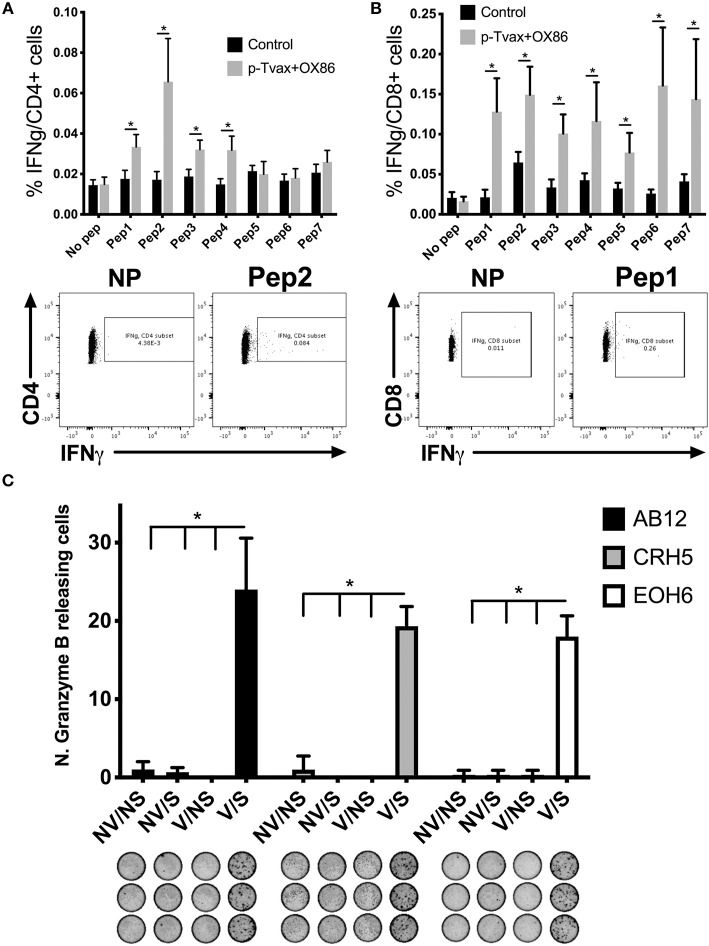
p-Tvax stimulates antigen-specific IFN-γ secreting T lymphocytes that produce Granzyme B when co-cultured with MM cells. BALB/c mice were vaccinated with two s.c. injections of p-Tvax peptides at days 2 and 9. CpG 1585 was used as adjuvant and injected at the same site of the p-Tvax peptides 2 days before vaccination, at days 0 and 7. OX40 agonist mAbs (OX86) were i.p. injected at days 4 and 9. Control mice were left untreated. Five days after the last vaccination, spleen cells were isolated and enumerated for assays. **(A)** Intracellular IFN-γ in CD4^+^ T cells was evaluated by flow cytometric ICS analyses of spleen cells cultured with or without p-Tvax peptides. Percentage of IFN-γ expressing CD4^+^ T cells are presented as mean ± S.E. Bottom Panel: Representative data from flow cytometric analysis of vaccinated and control mice. Lymphocytes were either unstimulated (no peptide = NP) or stimulated with peptide 2. CD4^+^ T cells were distinguished using a marker gate in the CD3 vs. CD4 dot plot. **(B)** IFN-γ in CD8^+^ T cells was evaluated in spleen cells cultured with or without p-Tvax peptides. Percentage of IFN-γ expressing CD8^+^ T cells are presented as mean ± S.E. Bottom Panel: Representative data from flow cytometric analysis of vaccinated and control mice. Lymphocytes were either not stimulated (NP) or stimulated with peptide 1. CD8^+^ T cells were distinguished using a marker gate in the CD3 vs. CD8 dot plot. For A and B, statistical significance between treated and control groups was determined by ANOVA followed by Bonferroni test (^*^p < 0.05, *n* = 5). **(C)** Secretion of Granzyme B was evaluated by ELISPOT assay. Spleen cells from vaccinated mice (V) or unvaccinated (no vaccine = NV) were activated with p-Tvax peptides in the presence of 5 IU/ml IL-2 for 6 d (stimulation = S) or incubated with no peptide (no stimulation = NS). Following 4 h of incubation with different MM cells (AB12, CRH5, EOH6), the number of spot-forming cells per 10^5^ cells was evaluated and results represented as mean ± S.E. Statistical differences between vaccinated mice, stimulated with peptides (V/S), and the other control conditions were evaluated by ANOVA followed by Bonferroni test (^*^p < 0.05, *n* = 3).

To determine if p-Tvax induces antigen-specific CD4^+^ and CD8^+^ T cells, we vaccinated BALB/c mice and evaluated T cell responses using intracellular cytokine staining (ICS). Cells from spleens of immunized mice were re-stimulated with each peptide listed in Table 1 and production of IFN-γ investigated with flow cytometry. In these assays, each of the p-Tvax peptides induced higher levels of IFN-γ^+^ CD8^+^ T cells compared to unstimulated CD8^+^ T cells, but only peptides 1, 2, 3, and 4 induced significant numbers of IFN-γ^+^ CD4^+^ T cells (Figures 2A,B).

We also confirmed that p-Tvax-induced T cells are able to recognize the antigen-specific epitopes on the surface of MM cells and initiate a Granzyme B-based cytolytic response. For these assays, we pooled the T cells stimulated with each peptide and evaluated their Granzyme B secretion by ELISPOT, following incubation with MM cells. Three different MM cell lines were used as target cells for these analyses. This included CRH5 and EOH6 cells, which are known to express the antigens included in p-Tvax, and AB12 cells, in which antigen expression was unknown at the time of the vaccine design. In these assays, we used effector T cells from unvaccinated mice as controls that, along with T cells from vaccinated mice, were unstimulated or stimulated with all the p-Tvax peptides. T cells from vaccinated mice stimulated with the p-Tvax peptides produced high numbers of Granzyme B spots when co-cultured with all 3 MM cell lines (Figure 2C). Importantly, even though the p-Tvax design was specific for the antigens expressed in CRH5 and EOH6 MM cells, this vaccine was able to elicit T cells that also recognized AB12 MM cells. This suggests that the antigens included in p-Tvax represent a group of commonly expressed proteins across mouse MM tumors. In contrast, Granzyme B ELISPOT experiments performed using non-tumor mesothelial cells did not show differences between unstimulated and stimulated T cells in both vaccinated and control mice (data not shown).

### Vaccination With p-Tvax and Engagement of the OX40 Receptor Elicits Potent Anti-tumor Responses in Subcutaneous and Intraperitoneal Mouse Models of MM

Multi-antigen cancer vaccines such as p-Tvax may serve as an effective approach to stimulate multiple populations of cancer-specific T cells, but the activity of these immune cells is hindered within the tumor microenvironment by several mechanisms, including the suppressive action by T regulatory cells (Tregs). Since OX40 agonists have been demonstrated to reduce the number of Tregs in the tumor microenvironment (10, 19), we evaluated the combination of p-Tvax with these agonistic antibodies as a therapeutic approach for MM in animal models.

**Figure 3:**
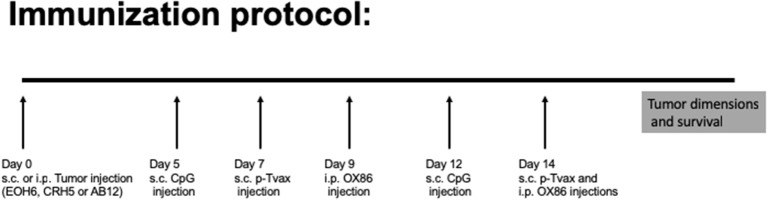
Experimental approach to evaluate p-Tvax and OX40 agonists anti-cancer properties. Two s.c. vaccinations were administered with p-Tvax separated by 1 week. Two days before each p-Tvax immunization, CpG adjuvant injections were performed. To engage the OX40 receptor on T cells, we performed two injections of the OX86 mAb, with the first dose injected 5 d prior to the last p-Tvax vaccination, and the second on the same day as the last p-Tvax injection. Untreated controls and mice treated with the different immunotherapies were followed for tumor dimension and survival.

**Figure 4:**
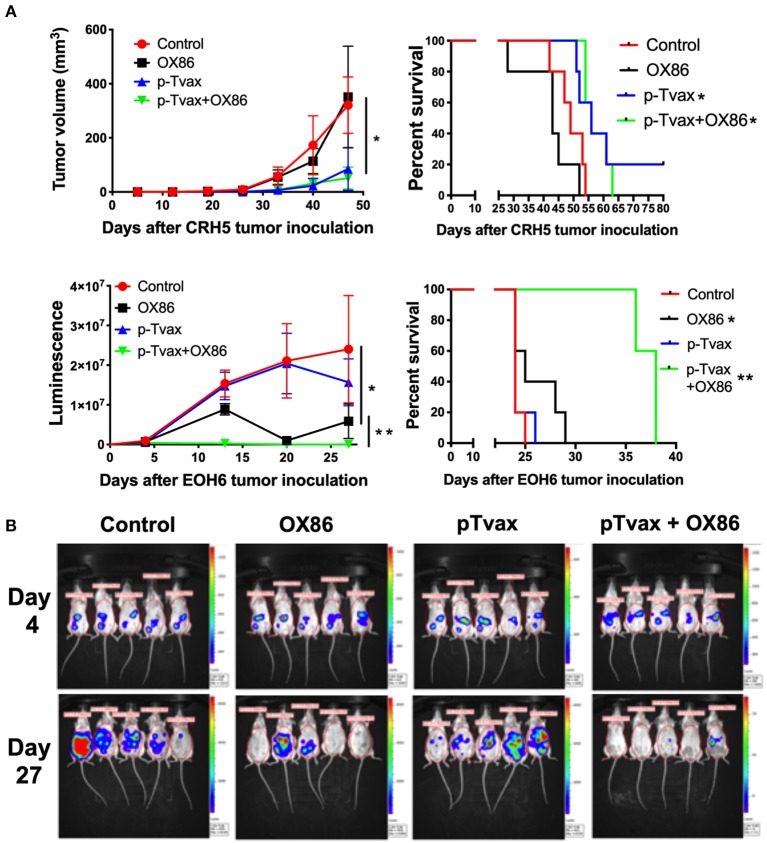
Combination of p-Tvax vaccine and OX40 agonists delays tumor growth and improves survival in subcutaneous and intraperitoneal mouse models of MM. **(A)** BALB/c mice were injected s.c. with 5 × 10^4^ CRH5 MM cells, or i.p. with 2 × 10^5^ EOH6 MM cells expressing luciferase. Seven and 14 d after tumor injection, mice were vaccinated with a s.c. injection of p-Tvax peptides. CpG adjuvant was injected at days 5 and 12, while 200 μg of OX86 was injected at day 9 and 14. Tumor volumes are showed on the left and animal survivals on the right for mice injected with CRH5 (Top) and EOH6 (Bottom). Tumor volumes were measured weekly with a caliper for s.c. tumors. I.p. MM dimensions were assessed by measuring luciferase activity with IVIS imaging following injection with luciferin substrate. Statistical significance between unvaccinated controls and single treatment (^*^) as well as between single and combination treatments (^**^), was determined by ANOVA followed by Bonferroni test (^*^p < 0.05, *n* = 5). For survival, mice were followed until s.c. CRH5 tumors reached volumes of 300 mm^3^ and were then sacrificed. In i.p. models with EOH6 MM cells, survival was assessed by euthanizing mice at first sign of morbidity. Log-rank analysis was used to determine significance between control and single treatment (^*^), and between single and combination treatments (^**^) (*p* < 0.05, *n* = 5). **(B)** Representative images from IVIS tumor dimension analysis of mice carrying EOH6 tumors, vaccinated with the different immunotherapies.

For the immunization protocol, we chose to administer two vaccinations with p-Tvax separated by 1 week. CpG adjuvant injections were performed 2 d before each p-Tvax immunization. This protocol elicits potent immune responses in mice (20). To engage the OX40 receptor on T cells, we performed two injections of the OX86 mAb, with the first dose injected 5 d prior to the last p-Tvax vaccination, and the second on the same day as the last p-Tvax injection (Figure 3). This protocol was chosen to minimize the clearance of the OX86 mAb by the immune system since these antibodies were developed in a species different from mice (i.e., rat). In s.c. models of MM, we used CRH5 MM cells and initiated vaccinations when tumors reached 3–4 mm in diameter. In these mice, we observed reduced tumor growth with p-Tvax alone or in combination with the OX86 mAb compared to unvaccinated controls or OX86 mAb alone. Survival analyses revealed that mice vaccinated with p-Tvax or with p-Tvax and OX86 mAb exhibited prolonged median survival compared with the controls or with OX86 mAb alone (Figure 4A). Since MM usually develops from mesothelial cells lining both thoracic and peritoneal cavities, we developed a clinically relevant MM model to test the therapeutic efficacy of p-Tvax and OX86 mAbs. For this model, we i.p.injected EOH6 cells previously transduced with a lentiviral vector encoding the luciferase enzyme, which enabled the localization and evaluation of tumor dimensions in live mice using an IVIS imaging system. In these mice, p-Tvax was less efficacious as indicated by reduced tumor dimensions closer to the end of the protocol (day 27) and differences over time were not statistically significant when compared with controls. In contrast, the OX86 mAb alone exerted statistically significant anti-cancer activity that delayed tumor growth for the entire protocol. These results were dramatically improved by the combination of p-Tvax and OX86 mAb, with mice showing no signs of tumor at day 20. In these mice, however, tumor cells were not completely eliminated and became visible with the IVIS machine the following week. Survival analysis of these mice showed that vaccination with p-Tvax together with the OX86 mAb resulted in prolonged survival compared with OX86 mAb alone. The p-Tvax vaccine alone did not lead to increased survival (Figure 4).

We also evaluated the anti-cancer effects of p-Tvax and the OX86 mAb in mice injected with AB12 MM cells. Results showed that p-Tvax reduced tumor growth, but did not improve survival. The OX86 mAb alone delayed tumors and improved survival compared to controls. The combination of p-Tvax plus the OX86 mAb was effective in delaying tumor growth equivalent to the monotherapy treatments. In survival experiments, p-Tvax in combination with OX86 mAbs dramatically improved overall survival, with 20% of vaccinated mice experiencing complete tumor regression. These results were statistically significant when compared with controls or each immunotherapy alone (Supplementary Figure 2). Importantly, no adverse events were observed for any treatment groups such as acute effects, distress, or weight loss, and gross tissue examination failed to uncover any toxicity in the organs (kidney, brain, spleen, liver, and lungs). It is important to mention that the expression of p-Tvax antigens was unknown in AB12 MM cells when these experiments were performed, but subsequently confirmed before the publication of this article (Supplementary Figure 2).

### Immunotherapy With p-Tvax Plus the OX86 mAb Induces an Immunostimulatory Tumor Microenvironment

**Figure 5:**
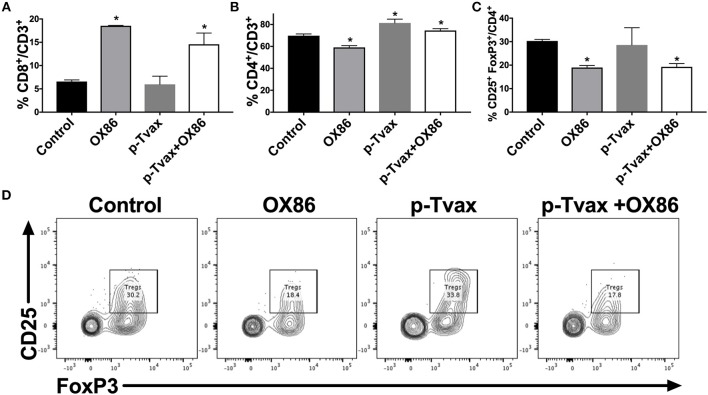
p-Tvax in combination with OX40 agonists induce CD8^+^ and CD4^+^ T cell tumor infiltration while reducing T regulatory cells. CRH5 tumors from controls or from mice treated with p-Tvax, OX86 mAbs, or with a combination of the two, were collected and enzyme-digested. Staining was performed using anti-mouse CD3, CD8, CD4, CD25, and FoxP3 antibodies with live cells distinguished from debris using LIVE/DEAD^®^ cell viability dye. **(A)** Percentage of CD8^+^ T cells in CRH5 tumors from treated and control mice. Results represent mean ± S.E. with means of each group compared using ANOVA followed by Bonferroni test (^*^p < 0.05, *n* = 5, vs. Control). Data for CD4^+^ T cells and Tregs cells are showed, respectively in **(B,C)**. **(D)** Representative data from flow cytometric analysis of CRH5 tumors in treated and control mice using CD25 and FoxP3 markers for Tregs, including Tregs that were previously gated in the CD3 vs. CD4 dot plot.

We next evaluated the effects of the different immunotherapies in MM models in terms of leukocyte cell populations in tumors using multicolor flow cytometry. This enabled the simultaneous identification of several different cell types in one sample, including CD8^+^ and CD4^+^ T cells, and Tregs. In CRH5 tumors, a higher percentage of CD4^+^ T helper cells was detected in p-Tvax vaccinated mice vs. controls, while abundance of CD8^+^ T cells and Tregs was not different from controls. The OX86 mAb increased abundance of CD8^+^ cytotoxic T cells, but reduced numbers of CD4^+^ T cells and Tregs. The combination of p-Tvax and the OX86 mAb modified the tumor microenvironment in a manner that led to more optimal anti-cancer immune conditions as supported by higher abundance of both CD8^+^ and CD4^+^ T cells, and reduced percentages of Tregs (Figure 5). In EOH6 tumors, we observed similar results with the only difference found with OX86 mAb that did not reduce significantly the number of CD4^+^ Tregs when used as single treatment (Supplementary Figure 3). To further characterize the tumor infiltrate, we also measured the expression of PD-1 in CD4^+^ and CD8^+^ T cells, as well as the expression of OX40 in Tregs. PD-1 expression was significantly reduced in CD4^+^ T cells following treatment with both p-Tvax and OX86 mAb, while its expression did not change in CD8^+^ T cells. Interestingly, the expression of OX40 in CD4^+^ CD25^+^ Tregs, which represent their activation status, was reduced following treatment with either OX86 mAb or with the combination of p-Tvax plus OX86 (Supplementary Figure 4).

## Discussion

In this study, we developed a novel immunotherapy combination comprised of a multi-antigen MM-specific vaccine combined with an OX40 agonist mAb leading to the reduction of tumor growth. The vaccine design started with the analysis of the MM tumor transcriptome performed in neoplastic tissues generated from two different MM cell lines, CRH5 and EOH6. Antigens that were highly expressed in both cells were chosen as candidates for inclusion in our vaccine. It has been established that the high expression of certain proteins within tumor cells leads to increased antigenic processing and presentation in the context of MHC-I and -II (21, 22). These fragments, referred to as tumor epitopes, represent targets for T cells that can specifically recognize the tumor cell for immune-mediated elimination. To minimize off-target effects that could lead to the development of autoimmunity, it is important to choose antigens that are not expressed or minimally detectable in normal tissues (23). In this regard, we also evaluated antigen expression in normal tissues in our laboratory as well as expression data from the literature (17, 18). This led to the selection of seven antigens found to be overexpressed in both mouse MM tumor lines under investigation and that were nearly absent in all other mouse normal tissues: KIF20A, KIF2C, MMP9, MNDA, OLFML2B, TROAP, and ULBP1. It is important to mention that we also tried to confirm the protein expression of these antigens in MM tumors using western blot. In these experiments, we evaluated several antibodies and specifically detected high levels of KIF20A, OLFML2B, and TROAP in MM tumors. Regarding the other proteins, the antibodies tested could not specifically recognize the antigens that we included in the vaccine. These negative results may be due to the rapid degradation of the proteins, or to their improper folding in tumor cells. However, it has been demonstrated that pre-maturely truncated or abnormally folded proteins are exported to the cytosol, processed by the proteasome and resultant peptides loaded on MHC-I and -II to trigger specific immune responses (24–26).

For each of the seven chosen antigen, we designed a peptide that contained both MHC-I and -II epitopes with the capacity to stimulate both CD8^+^ and CD4^+^ T cells. Epitope screening was performed using the iVAX platform which contains advanced *in silico* tools designed to identify highly immunogenic epitopes with EpiMatrix, while selecting against epitopes that may stimulate Treg or autoimmune responses with JanusMatrix. By including these optimized epitopes in our vaccine, we reduced the potential for suppressive or off-target effects. The development of these innovative *in silico* tools will increasingly provide the capacity for safe and effective vaccines (5–7).

Mice vaccinated with the multi-antigen vaccine (p-Tvax), mounted antigen-specific immune responses that involved both CD8^+^ and CD4^+^ T cells. These data were obtained using intracellular staining of peptide-pulsed T cells followed by flow cytometry analysis. A different approach to confirm these data, and to provide more quantitative results, would have been the characterization of antigen-specific T cells using MHC tetramers. Unfortunately, these reagents are not commercially available for the epitopes involved in this study. T cells from p-Tvax vaccinated mice were also demonstrated to recognize and attack tumor cells by secreting Granzyme B in ELISPOT assays performed with different MM cell lines. Among these MM cells recognized by vaccine-induced T cells was the AB12 cell line. AB12 cells were not included in the initial transcriptomic analyses for antigen selection, and in this manner served as an important comparison group in our studies. These data demonstrate that, by selecting multiple antigens commonly expressed in several MM cases, it is possible to construct an off-the-shelf vaccine that may be efficacious for a wide variety of MM. This approach to cancer vaccine development may complement or be used in place of personalized cancer vaccine approaches. In addition, off-the-shelf cancer vaccines bring the possibility of prophylactic immunization of individuals, which cannot be achieved with therapeutic personalized vaccines. Personalized immunizations are considerably more expensive than off-the-shelf vaccines due to of the cost of peptide synthesis (small vs. large production under GMP conditions). In addition, personalized vaccine epitopes tailored for each cancer patient's mutanome requiring sophisticated techniques such as whole-exome sequencing, RNA sequencing, and *in silico* immunogenicity prediction algorithms that are not as yet widely available. The safety profile of “off the shelf” vaccines such as the one proposed here can be ascertained well in advance of treatment, whereas the e safety profile of personalized anti-cancer vaccines remains to be fully evaluated. For example, a recent study showing that the number of somatic mutations in normal cells in cancer-related genes may be several times higher than in the cancer counterparts (27). Alternatively, vaccines such as the one described in this research study could be used in conjunction with personalized vaccines, to augment the number of epitopes to which the patients may respond.

In mouse models, p-Tvax as a stand-alone treatment showed limited efficacy in delaying tumor growth or in improving survival in mice carrying certain MM tumors. This was an expected outcome given the likelihood of immune suppression within the tumor environment. This is why efficacy of cancer vaccine can be enhanced by the inclusion of immune stimulatory Abs such as the OX40 agonist we chose for our study. In fact, the combination therapy using p-Tvax plus the OX40 agonist showed both decreased tumor volumes and increased survival for all three MM mouse models. Interestingly, the OX40 agonist alone was not effective in one animal model carrying CRH5 MM cells when used as a single agent. This is consistent with data obtained in clinical trials involving such immune checkpoint mAbs, in which clinical responses are often limited to a subset of patients (28, 29).

Analyses of tumor tissues in mice treated with the different immunotherapies produced results that may be best explained when considering the combination treatment as a sum of all the benefits induced by each therapeutic approach, namely, the combined increase in CD4^+^ T cells induced by p-Tvax and the increase in CD8^+^ T cells induced by OX40 agonists. Further analysis of the tumor infiltrate also showed that the combination of p-Tvax and OX40 agonists produced other effects that may favor tumor clearance such as the reduced expression of the T cell exhaustion marker PD-1 in CD4^+^ T cells and the decrease in number of Tregs. It is important to point out that mice treated with the combination of p-Tvax and OX40 agonists also showed reduced expression of OX40 in intra-tumor Tregs, which indicates a lower regulatory activity of these cells and a more favorable prognosis (10, 30, 31). Our data collectively suggest that this combination of immunotherapies could be an attractive therapeutic strategy for MM patients. Also, considering that humanized OX40 agonists have already been developed and successfully passed the phase I clinical trials (13), this immunotherapeutic mAb approach may serve as an effective adjuvant to include along with cancer vaccines. We have demonstrated proof-of-concept supporting p-Tvax in human immunization, in which target antigens can be chosen from transcriptome studies performed in MM patients (32, 33). This may provide the framework for a new approach to combine this broadly acting vaccine with immunotherapy and/or personalized cancer vaccines, for treating not only MM, but other types of cancers as well.

## Ethics Statement

Animal experiments were performed in accordance with institutional guidelines and approved by the University of Hawaii IACUC (#16-2355).

## Author Contributions

PRH, LM, AD, and PB conceived of the idea. PRH, LN, AS, AW, and PB designed the methods with feedback from the others. PB implemented the methods and collected the results. ES, MP, and AS prepared the vaccines included in this study. TP, GC, TF, FH, and PB performed animal studies and T cell analyses. GR, PH, LM, and AD identified T cell epitopes using JanusMatrix algorithm. MM, VK, and YD analyzed the transcriptome data and performed statistical analyses. PRH, AD, and PB wrote the paper. All authors read and approved the final manuscript.

### Conflict of Interest Statement

AD and LM are employees of EpiVax, a vaccine and therapeutic design company, and AD is majority stockholders. AW is President/CSO of, has received commercial research funding from, and has ownership interest in AgonOx. These authors recognize the presence of potential conflicts of interest and affirm that the information represented in this paper is original and unbiased observations. The remaining authors declare that the research was conducted in the absence of any commercial or financial relationships that could be construed as a potential conflict of interest.
