# Supplementary material for: Multi-antigen Vaccination With Simultaneous Engagement of the OX40 Receptor Delays Malignant Mesothelioma Growth and Increases Survival in Animal Models
Source: Front Oncol. 2019 Aug 2;9:720. doi: 10.3389/fonc.2019.00720 (PMC6688537; doi:10.3389/fonc.2019.00720)
Supplement: Supplementary file 1 [file Data_Sheet_1.docx]

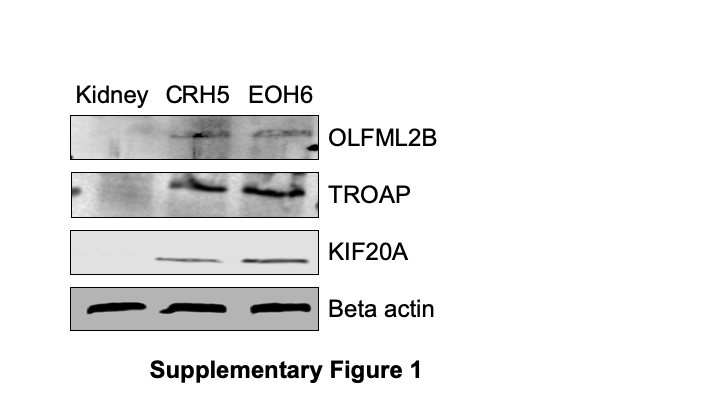


**Supplementary Figure 1. CRH5 and EOH6 MM tumors overexpress 3 of the 7 antigens targeted by p-Tvax.** Western blot analysis was performed with lysates from CRH5 and EOH6 tumors, compared with normal kidney lysate. Antibodies for OLFML2B, TROAP and KIF20A detected specifically the proteins targeted by p-Tvax. Antibodies for KIF2C, MMP9, MNDA and ULBP1 were also tested, but provided unspecific results.


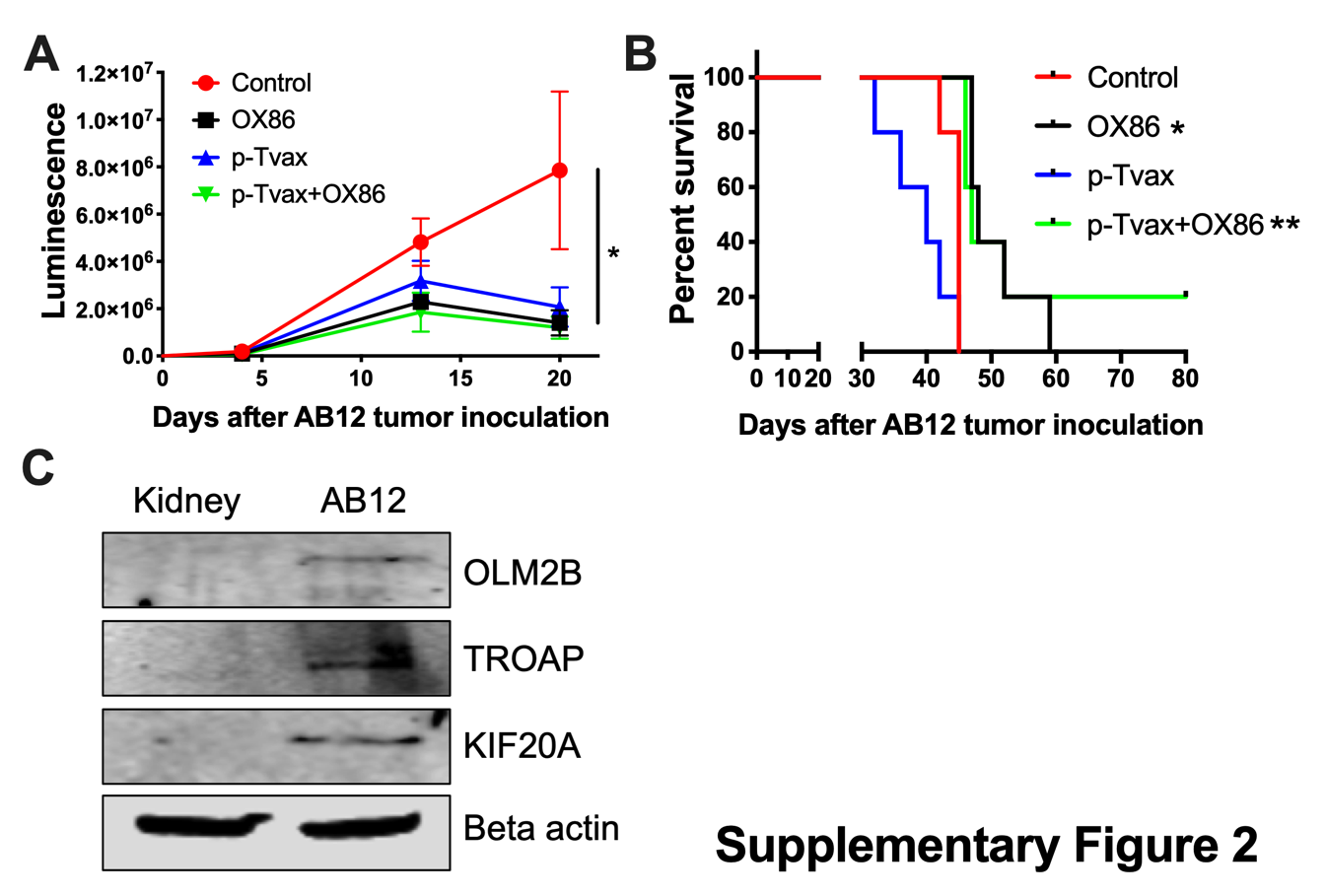


**Supplementary Figure 2. Combination of p-Tvax vaccine and OX40 agonists delays tumor growth and improves survival in AB12 intraperitoneal mouse models of MM.** BALB/c mice were injected i.p. with 5 X 10^4^ AB12 MM cells expressing luciferase. Seven and 14 days after tumor injection, mice were vaccinated with a s.c. injection of p-Tvax peptides. CpG adjuvant was injected at days 5 and 12, while 200 µg of OX86 was injected at day 9 and 14. Tumor volumes are showed on A and animal survivals on B. MM dimensions were assessed by measuring luciferase activity with IVIS imaging following injection with luciferin substrate. Statistical significance between unvaccinated controls and the different treatments was determined by ANOVA followed by Bonferroni test (**p*<0.05, n=5). Survival was assessed by euthanizing mice at first sign of morbidity. Log-rank analysis was used to determine significance between control and single treatment (*), and between single and combination treatments (**) (*p*<0.05, n=5). C) Western blot analysis of AB12 tumors vs normal Kidney for 3 of the 7 antigens targeted by p-Tvax.


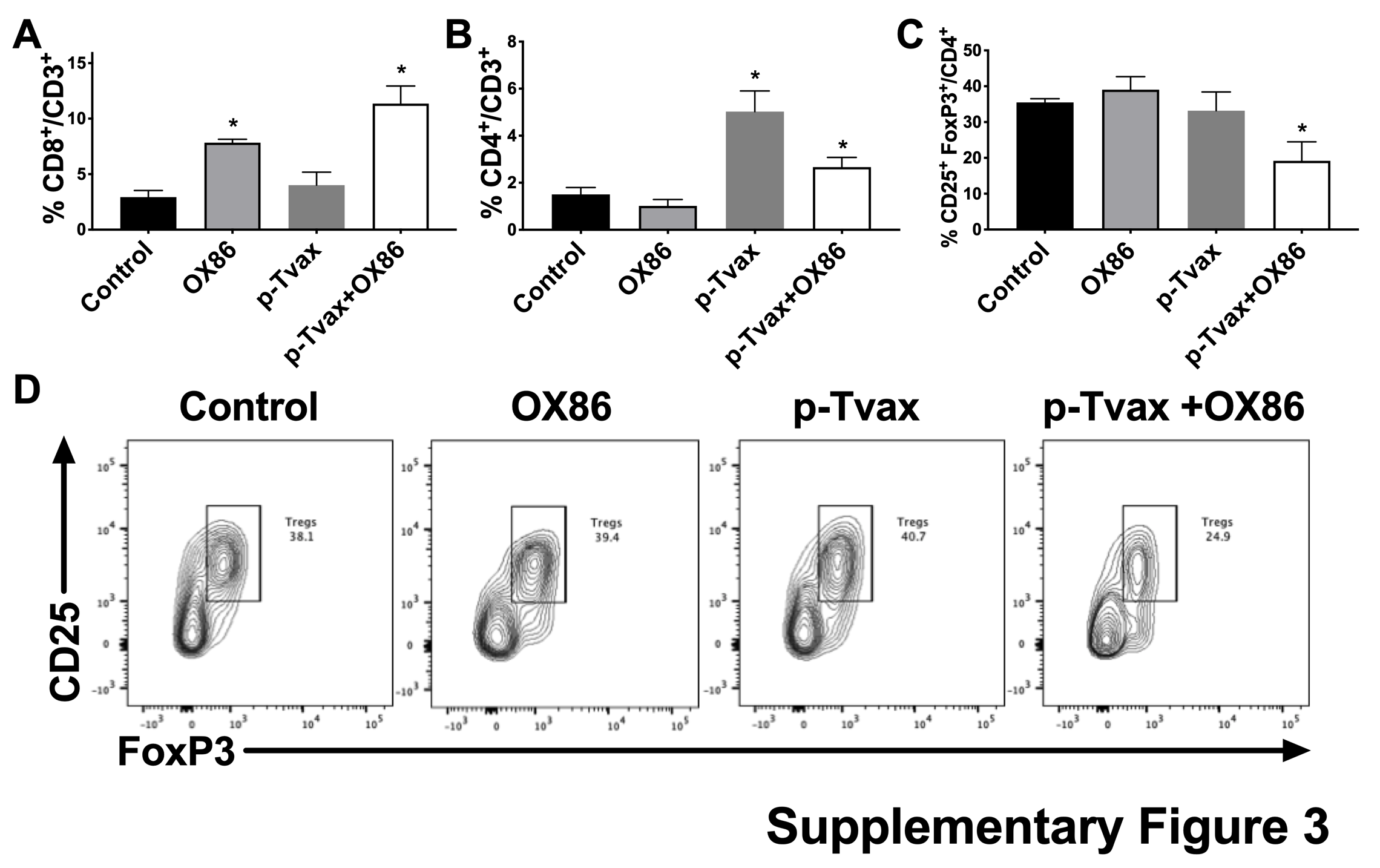


**Supplementary Figure 3. p-Tvax in combination with OX40 agonists induce CD8^+^ and CD4^+^ T cell tumor infiltration while reducing T regulatory cells.** EOH6 tumors from controls or from mice treated with p-Tvax, OX86 mAbs, or with a combination of the two, were collected and enzyme-digested. Staining was performed using anti-mouse CD3, CD8, CD4, CD25 and FoxP3 antibodies with live cells distinguished from debris using LIVE/DEAD^®^ cell viability dye. A) Percentage of CD8^+^ T cells in EOH6 tumors from treated and control mice. Results represent mean ± S.E. with means of each group compared using ANOVA followed by Bonferroni test (**p*<0.05, n=5, Vs. Control). Data for CD4^+^ T cells and Tregs cells are showed respectively in (B) and (C). D) Representative data from flow cytometric analysis of EOH6 tumors in treated and control mice using CD25 and FoxP3 markers for Tregs, including Tregs that were previously gated in the CD3 vs. CD4 dot plot.


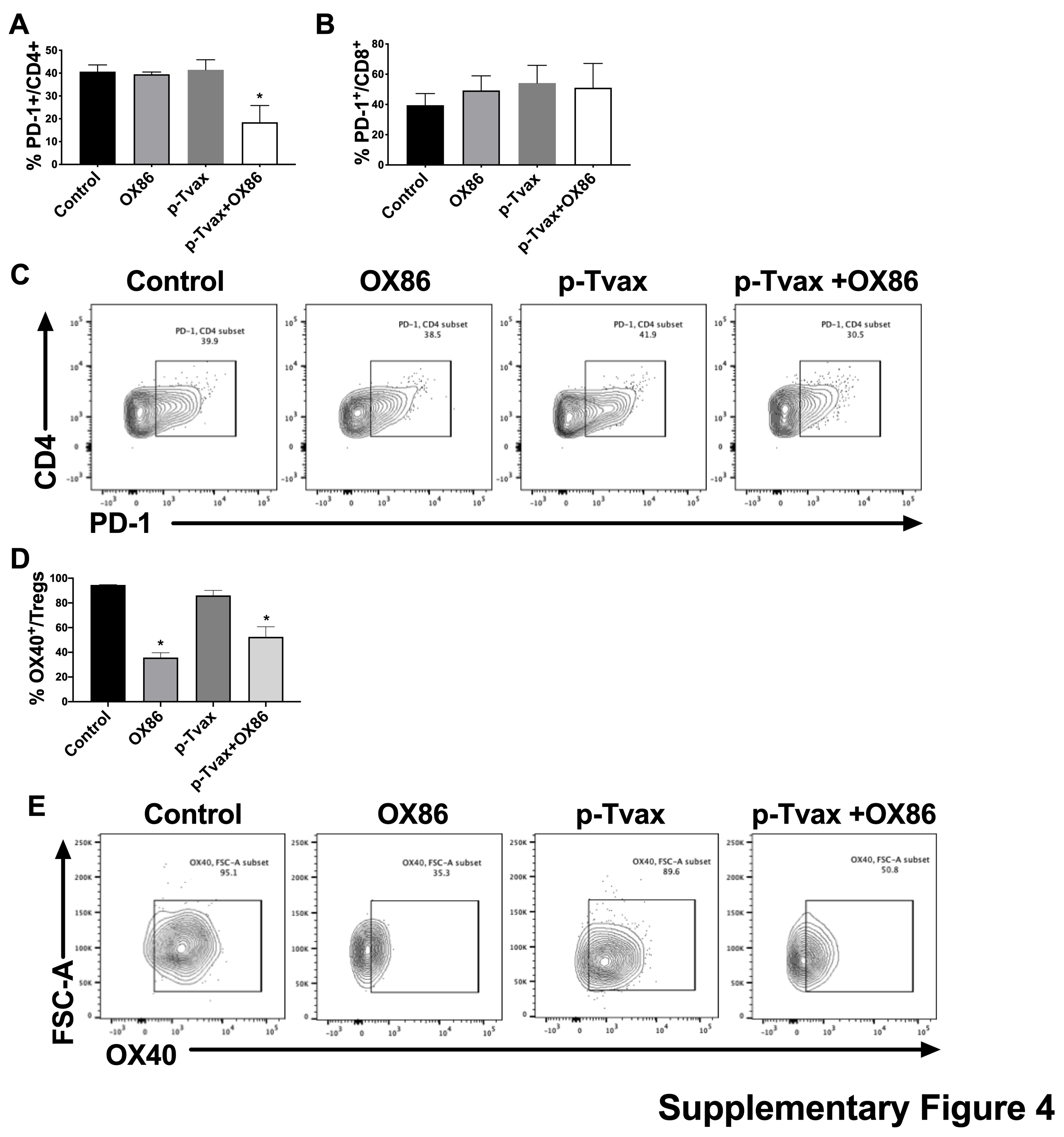


**Supplementary Figure 4. p-Tvax in combination with OX40 agonists reduces the expression of PD-1 in CD4^+^ T cells, and the expression of OX40 in Tregs.** EOH6 tumors from controls or from mice treated with p-Tvax, OX86 mAbs, or with a combination of the two, were collected and enzyme-digested. Staining was performed using anti-mouse CD3, CD8, CD4, PD-1 and OX40 antibodies with live cells distinguished from debris using LIVE/DEAD^®^ cell viability dye. Percentages of PD-1^+^/CD4^+^ T cells in EOH6 tumors from treated and control mice are showed in (A), while percentages of PD-1^+^/CD8^+^ T cells are showed in (B). These results represent mean ± S.E. with means of each group compared using ANOVA followed by Bonferroni test (**p*<0.05, n=5, Vs. Control). C) Representative data from flow cytometric analysis of EOH6 tumors in treated and control mice using CD4 and PD-1 markers, with T cells previously gated in the CD3 vs. FSC-A dot plot. D) Percentages of OX40^+^ Tregs in EOH6 tumors from treated and control mice Results are represented as mean ± S.E. with means of each group compared using ANOVA followed by Bonferroni test (**p*<0.05, n=5, Vs. Control). E) Representative data from flow cytometric analysis of EOH6 tumors in treated and control mice using OX40 mAb, with CD4^+^ Tregs previously gated in the CD25 vs. FoxP3 dot plot.
